# Supplementary material for: Time-resolved parameterization of aperiodic and periodic brain activity
Source: eLife. 2022 Sep 12;11:e77348. doi: 10.7554/eLife.77348 (PMC9467511; doi:10.7554/eLife.77348)
Supplement: Transparent reporting form [file elife-77348-transrepform1.pdf]

eLIFE
1st Floor
24 Hills Road
Cambridge CB2 1JP, UK
P 01223 855340
W elifesciences.org
T @elife

# *eLife's* transparent reporting form

We encourage authors to provide detailed information *within their submission* to facilitate the interpretation and replication of experiments. Authors can upload supporting documentation to indicate the use of appropriate reporting guidelines for health-related research (see EQUATOR Network), life science research (see the BioSharing Information Resource), or the ARRIVE guidelines for reporting work involving animal research. Where applicable, authors should refer to any relevant reporting standards documents in this form.

If you have any questions, please consult our Journal Policies and/or contact us: editorial@elifesciences.org.

**Sample-size estimation**

- You should state whether an appropriate sample size was computed when the study was being designed
- You should state the statistical method of sample size computation and any required assumptions
- If no explicit power analysis was used, you should describe how you decided what sample (replicate) size (number) to use

Please outline where this information can be found within the submission (e.g., sections or figure legends), or explain why this information doesn't apply to your submission:

| |
|---|
| Sample size was determined by the amount of data available from the open repositories (i.e., LEMON; Babayan et al., 2019; https://openneuro.org/datasets/ds000221/versions/00002 and Mizuseki et al. 2009; CRCNS.org. doi:10.6080/K09G5JRZ). |

**Replicates**

- You should report how often each experiment was performed
- You should include a definition of biological versus technical replication
- The data obtained should be provided and sufficient information should be provided to indicate the number of independent biological and/or technical replicates
- If you encountered any outliers, you should describe how these were handled
- Criteria for exclusion/inclusion of data should be clearly stated
- High-throughput sequence data should be uploaded before submission, with a private link for reviewers provided (these are available from both GEO and ArrayExpress)

Please outline where this information can be found within the submission (e.g., sections or figure legends), or explain why this information doesn't apply to your submission:

| |
|---|
| We do not perform any DNA sequencing or biological replicates in our study. This not applicable to our methods research in electrophysiology.<br><br>We provide detailed information concerning participant inclusion and exclusion when applicable (i.e., Study 2: Resting-state electrophysiology data) in the respective methods section. We did not exclude any other participants or data otherwise. |

eLife Sciences Publications, Ltd is a limited liability non-profit non-stock corporation incorporated in the State of Delaware, USA, with company number 5030732, and is registered in the UK with company number FC030576 and branch number BR015634 at the address 1st Floor, 24 Hills Road, Cambridge CB2 1JP | August 2014

**Statistical reporting**

- Statistical analysis methods should be described and justified
- Raw data should be presented in figures whenever informative to do so (typically when N per group is less than 10)
- For each experiment, you should identify the statistical tests used, exact values of N, definitions of center, methods of multiple test correction, and dispersion and precision measures (e.g., mean, median, SD, SEM, confidence intervals; and, for the major substantive results, a measure of effect size (e.g., Pearson's r, Cohen's d)
- Report exact p-values wherever possible alongside the summary statistics and 95% confidence intervals. These should be reported for all key questions and not only when the p-value is less than 0.05.

Please outline where this information can be found within the submission (e.g., sections or figure legends), or explain why this information doesn't apply to your submission:

We provide a detailed explanation of all the statistical tests we run in the relevant methods section and provide the code we used to compute these tests.

We present raw data whenever possible (Figure 3, Figure 4, Figure 3 – figure supplement 3, Figure 5 – figure supplement 1). For all plots, when applicable, we report the measure of dispersion in the figure captions.

We report exact p values in the Regression tables provided in the supplemental materials. We additionally report Bayes Factors in the text and supplemental materials.

See table of statistical tests attached.

(For large datasets, or papers with a very large number of statistical tests, you may upload a single table file with tests, Ns, etc., with reference to sections in the manuscript.)

**Group allocation**

- Indicate how samples were allocated into experimental groups (in the case of clinical studies, please specify allocation to treatment method); if randomization was used, please also state if restricted randomization was applied
- Indicate if masking was used during group allocation, data collection and/or data analysis

Please outline where this information can be found within the submission (e.g., sections or figure legends), or explain why this information doesn't apply to your submission:

This is not applicable to our experiments. We did not randomly assign participants or samples to groups.

**Additional data files ("source data")**

- We encourage you to upload relevant additional data files, such as numerical data that are represented as a graph in a figure, or as a summary table
- Where provided, these should be in the most useful format, and they can be uploaded as "Source data" files linked to a main figure or table
- Include model definition files including the full list of parameters used
- Include code used for data analysis (e.g., R, MatLab)
- Avoid stating that data files are "available upon request"

eLife Sciences Publications, Ltd is a limited liability non-profit non-stock corporation incorporated in the State of Delaware, USA, with company number 5030732, and is registered in the UK with company number FC030576 and branch number BR015634 at the address 1st Floor, 24 Hills Road, Cambridge CB2 1JP | August 2014

Please indicate the figures or tables for which source data files have been provided:

We have provided all the codes necessary to generate the data in our manuscript, analyses, and figures. We have additionally provided the simulations we produced on our OSF repository (in plain-text or csv format) and links to the open datasets we analyzed in our experiments (see Data Availability section).

Source data for figures (and figure supplements) has been provided where applicable (violin plots, histograms, scatter plots; in csv format). Specifically: Figure 2, Figure 3, Figure 4, Figure 2 – figure supplement 2, Figure 2 – figure supplement 3, Figure 2 – figure supplement 4, Figure 3 – figure supplement 2, Figure 3 – figure supplement 3, and Figure 5 – figure supplement 2. Source data for Figure 2 and its supplements was stored in our open-access OSF repository due to its large file size.

Summary Table of statistical tests

| Hypothesis | Name of test | N | Multiple test correction | Dispersion measure | Effect size |
|---|---|---|---|---|---|
| Predicting condition from specparam | Logistic regression | 147 observations from eyes-open, 176 observations from eyes-closed (total of 323 observations) | None / NA | 95% confidence intervals for betas in regression tables and SE in text. | We report the Log odds of the effect in Table 1 |
| Predicting condition from SPRiNT | Logistic regression | 177 participants with 2 observations, 1 participant with 1 observation (total of 355 observations) | None / NA | 95% confidence intervals for betas in regression tables and SE in text. | We report the Log odds of the effect in Table 2 and 3 |
| Predicting condition from wavelets | Logistic regression | 178 participants with 2 observations each (total of 356 observations) | None / NA | 95% confidence intervals for betas in regression tables and SE in text. | We report the Log odds of the effect in Table 4 |
| Predicting age group from SPRiNT | Logistic regression | 178 participants (177 for eyes-open) | None / NA | 95% confidence intervals for betas in regression tables and SE in text. | We report the Log odds of the effect in Tables 5 and 6 |
| Predicting age from short-time Fourier transform | Logistic regression | 178 participants | None / NA | 95% confidence intervals for betas in regression tables and SE in text. | We report the Log odds of the effect in Tables 7 and 8 |

| | | | | | |
|---|---|---|---|---|---|
| Predicting age from specparam | Logistic regression | 147 participants for eyes-open, 176 participants for eyes-closed | None / NA | 95% confidence intervals for betas in regression tables and SE in text. | We report the Log odds of the effect in Tables 9 and 10 |
| Predicting age group from wavelets | Logistic regression | 178 participants | None / NA | 95% confidence intervals for betas in regression tables and SE in text. | We report the Log odds of the effect in Tables 11 and 12 |
| Linear regression of model fit error with number of simulated peaks present | Linear regression | 1 150 000 (10 000 simulations x 115 time bins) | None / NA | 95% confidence intervals for betas and SE in text. | We report R-squared in the main text |
| Linear regression of aperiodic exponent with movement speed about transitions in locomotor behaviour | Linear regression | EC012 transition to rest: 1054; EC012 transition to movement: 1377; EC013 transition to rest: 5151; EC013 transition to movement: 4318. | None / NA | 95% confidence intervals for betas and SE in text. | We report R-squared in the main text |
| Comparing SPRiNT model fit error between condition | Two-sample t-test | 178 participants with 2 observations each (total of 356 observations) | None / NA | 95% confidence intervals of mean difference in text. | We report Cohen's *d* in Supplemental Materials |
